# Preparation and application of a thidiazuron·diuron ultra-low-volume spray suitable for plant protection unmanned aerial vehicles

**DOI:** 10.1038/s41598-021-84459-4

**Published:** 2021-03-02

**Authors:** Qin Liu, Kun Wei, Liyun Yang, Weiming Xu, Wei Xue

**Affiliations:** 1 State Key Laboratory Breeding Base of Green Pesticide and Agricultural Bioengineering, Key Laboratory of Green Pesticide and Agricultural Bioengineering, Ministry of Education, Center for Research and Development of Fine Chemicals, Guizhou University, Guiyang, 550025 China; 2 Renhuai Agricultural and Rural Bureau, Guizhou, 564500 Renhuai China

**Keywords:** Pharmaceutics, Field trials, Agroecology, Medicinal chemistry, Plant sciences

## Abstract

Spraying of defoliant can promote centralized defoliation of cotton and advance maturity to facilitate harvesting. Modern pesticide application equipment includes plant protection unmanned aerial vehicles (UAVs), which are used widely for spraying defoliants. However, commonly used defoliant formulations are mainly suspension concentrates and water-dispersible granules, which need to be diluted with water when used. These are not suitable for plant protection UAVs with limited load capacity, especially in arid areas such as Xinjiang, China. Therefore, we prepared a thidiazuron·diuron ultra-low-volume (ULV) spray, which can be used directly without dilution in water. We found that ULV sprays had better wettability than the commercially available suspension concentrate, could quickly wet cotton leaves and spread fully. The volatilization rate was lower. ULV sprays also showed better atomization performance and more uniform droplet distribution than the commercially available suspension concentrate. At a dosage of 4.50–9.00 L/ha, the coverage rate on cotton leaves was 0.85–4.15% and droplet deposition densities were 15.63–42.57 pcs/cm^2^; defoliation rate and spitting rate were also greater than those of the reference product. This study could be contributed to the development of special pesticide formulations suitable for UAVs.

## Introduction

Cotton is an important economic crop. As the main raw material of the textile industry, it plays a crucial role in China's national economy^1–3^. Xinjiang is a prominent planting base for cotton in China. For a long time, farmers relied on manual harvesting of cotton; however, the huge labor expenditure seriously reduced the income of the cotton farmers^4^. Therefore, machine-picking has become an inevitable choice for cotton harvesting because it reduces labor and planting costs^5^. However, application and popularization of mechanical picking in China is far from sufficient. The main problem is that cotton bolls do not open uniformly at harvest time, and the leaves do not fall off completely, leading to a high content of impurities in mechanically picked cotton. Therefore, spraying defoliant to make leaves fall off and promote earlier and uniform opening of cotton bolls is an important step in accelerating the process of mechanized cotton picking and improving the quality of cotton seeds^6–8^.

Thidiazuron is a urea plant growth regulator that promotes the production of ethylene and abscisic acid in cotton and inhibits the transport of auxin. A separation layer forms between the leaf and the petiole to promote leaf shedding^9,10^. Combined use of thidiazuron and diuron produces better defoliation effects than thidiazuron alone, with diuron enhancing defoliation activity especially in low-temperature environments^11^. Ethylene is a gaseous hormone that promotes fruit ripening and shedding of old leaves and other organs^12,13^. Defoliation of cotton plants is closely related to the ripening and opening of cotton bolls, and a combination of defoliant and ethephon improves the effect of defoliation. Ethephon is a type of ethylene precursor; after being absorbed by cotton, it promotes the biosynthesis of ethylene, resulting in ripening and cracking of cotton bolls^14^. Therefore, chemical defoliation and ripening are essential for machine picking of cotton. Defoliation stimulates cotton bolls to open relatively early and uniformly, reduces impurities in seed cotton, avoids pollution of cotton wool with dead leaf debris, and improves the quality and yield of cotton, thereby increasing the planting efficiency of cotton.

The method used for spraying defoliants plays an important role in the efficiency of cotton defoliation. Defoliants are generally used during the boll-opening stage^15–17^. Improving the coverage of defoliants on cotton plants is key to improving the efficacy of defoliants. Currently, cotton growers in China’s Xinjiang region mainly use boom-type sprayers to spray pesticides. Compared with knapsack sprayers, these have wide spray width, large capacity, and high efficiency, leading to savings in labor costs and time. However, the terrain can restrict boom-type sprayers, which can damage plants, spraying can be uneven, and their efficacy is not excellent^18^. In addition, given the large application volumes, the effective utilization rate of pesticides is low, potentially causing pollution^19^.

Therefore, in recent years, cotton growers in China’s Xinjiang region have begun to spray defoliants using plant protection unmanned aerial vehicles (UAVs)^20^. Compared with traditional mechanical and artificial pesticide application, use of UAVs is labor and time-saving, with the same significant effect on defoliation and ripening of cotton, but no significant effect on yield and quality^21^. With the introduction of UAVs, cotton cultivation has become more mechanized and technological. Pesticides sprayed by plant protection UAVs show high levels of safety and cause little harm to the human body. The UAVs produce a low-altitude and low-volume flight spray. The airflow generated by the propeller causes the pesticide mist to flow through the cotton plant from top to bottom with strong penetration, less drift, and uniform spray^22–24^. Therefore, the coverage rate of pesticide formulations is higher and more efficient than that using more traditional methods^25^. At the same time, UAVs can reduce land pollution caused by pesticides and improve pesticide utilization rates. The spray efficiency and effectiveness of plant protection UAVs are significantly better than those of boom-type sprayers, but there are few pesticide formulations suitable for plant protection UAVs. The low-altitude, small-volume spray produced by plant protection UAVs imposes stricter requirements on pesticide formulations^26–29^. Defoliants currently registered in China are mainly suspension concentrates, water-dispersible granules and wettable powders, which are not suitable for spraying using plant protection UAVs. This lack of pesticide formulations suitable for UAVs is the biggest obstacle in the development of UAV application. Ultra-low-volume pesticide sprays, with high-boiling-point oil solvent as carrier, have low volatility and strong adhesion. Compared with conventional pesticide formulations, they provide special advantages, such as high levels of coverage and better efficacy, making them ideal for plant protection UAVs^30^.

In this study, we prepared a thidiazuron·diuron ultra-low-volume spray suitable for use with plant protection UAVs. The wettability, atomization, and deposition performance of the ultra-low-volume spray on cotton leaves and its defoliation and ripening effects were studied.

## Experimental

### Materials

Unless otherwise noted, all chemicals were purchased from commercial suppliers and were used without further purification: Thidiazuron (95%), diuron (97%), chlorinated paraffin, and methyl oleate (Aladdin BioChem Technology Co., Ltd, China); Anionic adjuvant and nonionic adjuvant (BASF SE, Germany, 99%); Thidiazuron and diuron suspension concentrate (540 g/L) (Jiangsu Changqing agrochemical Co., Ltd., China).

### Screening of solvents

Different organic solvents were added to 0.2 g thidiazuron and 0.1 g diuron. After shaking uniformly, samples were heated in a water bath and subjected to ultrasonic oscillation to dissolve them. Since heating increases the volatilization of organic solvents, the temperature of the water bath did not exceed 35 °C, and the time of ultrasonic oscillation did not exceed 20 min. After this time, the dissolution state of the original pesticide was observed and the dissolution effects of different solvent combinations were compared.

### Screening of adjuvant

Adjuvants in the ultra-low-volume spray can improve the physical and chemical properties of the formulation and enhance its wetting and spreading properties^31^. Based on solvent screening, anionic and nonionic adjuvants were selected to build a stable pesticide loading system. The ultra-low-volume spray used a high-boiling-point oily solvent as a low-volatility carrier to facilitate the deposition of small droplets^32^. Methyl oleate is a methylated vegetable oil adjuvant with good biodegradability and low toxicity, and this was added as the oily solvent. Characteristics of an ultra-low-volume spray are low dosage and high concentration^33^. Droplets produced by spraying are extremely fine and easily drift, causing phytotoxicity to non-target organisms. It is necessary to add fillers to increase the viscosity and density of the formulation, reducing the risk of drift and phytotoxicity. Therefore, chlorinated paraffin was used as the filler at a proportion of 25%.

### Preparation of the ultra-low-volume spray oil concentrate

Thidiazuron and diuron were placed into a 250 mL beaker, then different organic solvents were added and stirred at 200 rpm for 20 min. After dissolving completely, the anionic adjuvant was added and stirred until the system was clear. Next, the nonionic adjuvant was added and stirred. After mixing to uniformity, chlorinated paraffin and methyl oleate were added sequentially, followed by stirring at 250 rpm for 25 min. The resulting pale yellow, single transparent, homogeneous liquid containing thidiazuron and diuron was used as an ultra-low-volume spray.

### Wettability measurement

The surface tension of each sample was measured using a SFZL-A1 surface tensiometer using the platinum plate method^34,35^. The contact angle of each sample on cotton leaves was measured using a JC2000D contact angle meter^36,37^. The viscosity of the ultra-low-volume sprays was measured using a NDJ-5S rotary viscometer. The reference product was 540 g/L thidiazuron·diuron suspension concentrate, which was recorded as 6. One gram of the reference product was diluted to 540 mL with water for subsequent experiments.

### Volatilization rate measurement

The volatilization rate of samples was determined using the filter paper suspension method^38,39^. The filter paper was weighed and recorded as m_1_. Approximately 1.0 mL of each sample was distributed evenly onto separate filter paper circles, and the weight was recorded as m_2_. Subsequently, the filter paper was suspended in a drying oven at 25 °C for 20 min. The filter paper was reweighed and recorded as m_3_. The volatilization rate was calculated according to Eq. (1):

1 $$ {\text{Volatilization rate }}\left( \% \right) \, = \frac{{{\text{m}}_{2} - {\text{m}}_{3} }}{{{\text{m}}_{2} - {\text{m}}_{1} }} \times 100{\text{\% }}{.} $$

### Atomization performance measurement

An indoor spraying device was used to simulate field application using the plant protection UAV. A spray particle size analyzer was used to test the atomization performance of the thidiazuron·diuron ultra-low-volume (ULV) spray and to analyze the effect of viscosity on droplet size and particle size distribution.

The indoor spraying device consisted of a DC voltage stabilized power supply, a peristaltic pump, and a rotary atomizer. The current of the DC voltage stabilized power supply was set to 1.00 A, and the voltage was adjusted to the range of 12–24 V. A laser tachometer was used to test the rotation speed of the rotary atomizer at various voltages. To analyze the stability of the rotary atomizer, a linear fitting of voltage and rotation speed was performed. The discharge of the peristaltic pump was set to 100 mL/min, and water was used as a spray. A spray particle size analyzer was used to test the size and distribution of the droplets ejected by the rotary atomizer at high-speed rotation. By analyzing the relationship between droplet size and the rotation speed of the rotary atomizer, the conditions for producing uniformly distributed droplets were found. Samples were sprayed according to the optimized conditions. Cumulative volume diameter and distribution information were collected to analyze the effect of formulation viscosity on atomization performance. The droplet size distribution (Rs) was calculated according to Eq. (2):

2 $$ Rs = \frac{{\text{D}}_{90} - {\text{D}}_{10}}{{{\text{D}}_{50}}}. $$

### Efficacy trial

In September 2017, an efficacy trial of the thidiazuron·diuron ultra-low-volume spray was carried out at the cotton planting base of Xinjiang Agricultural University in Shawan County (44° 19′ 31.92″ E, 85°37′ 0.88″ N), Xinjiang Uygur Autonomous Region, China. The deposition effect of the droplets, defoliation rate, and cotton boll opening rate were tested under different application dosages. The height of cotton plants was approximately 1 m. On the day of the test, the temperature was 13–27 °C and the wind force was ≤ 3.

The application equipment used in the efficacy trial was a XAG P-20 plant protection UAV (XPLANT Co., Ltd., China). Five treatments were set up. Treatments 1–4 were nebulized thidiazuron·diuron ultra-low-volume spray prepared in this study, corresponding application dosages were 4.50, 6.00, 7.50, and 9.00 L/ha. Treatment 5 was 54% thidiazuron·diuron suspension concentrate. The area of each trial was 9 × 100 m^2^.

### Deposition measurement

A droplet test card (oil-sensitive paper/water-sensitive paper, 2.6 cm × 7.6 cm) was used to test the effect of droplet deposition in the field when the formulation was sprayed by the plant protection UAV. The width of each measurement area was 9 m, which was suitable for the UAV to fly across three times. Three points were randomly selected from each of the three flight paths, and nine sampling points were investigated. A test card was fixed using a paper clip to the cotton plant canopy at each sampling point to collect droplets. At the end of the experiment, the droplet test cards were scanned using a scanner. Image-pro Plus software was used to analyze droplet size, droplet coverage and droplet deposition density on the test cards, and droplet distribution uniformity was calculated.

### Defoliation rate and boll opening rate measurement

The effect of different treatments was tested by investigating the defoliation rate and boll opening rate of cotton plants in each treatment plot. For each plot, five sampling points in the shape of an "X" were randomly selected; at each point, five cotton plants were randomly selected as survey points. The total number of leaves, bolls, and opening bolls of cotton plants were counted before and 15 days after spraying the pesticide. The defoliation rate and boll opening rate were calculated according to Eqs. (3) and (4):

3 $$ {\text{Defoliation rate }} = \frac{{{\text{M}}_{1} - {\text{M}}_{2} }}{{{\text{M}}_{1} }} \times 100{\text{\% ,}} $$

4 $$ {\text{Boll opening rate }} = \frac{{{\text{N}}_{2} }}{{{\text{N}}_{1} }} \times 100{\text{\% }}{.} $$

*M*_*1*_ and *M*_*2*_ are the number of cotton leaves before and after application; *N*_*1*_ is the total number of bolls of cotton plants, *N*_*2*_ is the number of opening bolls.

### Ethics approval

Ethics approval is not required for this paper.

## Results and discussions

### Screening of solvent and adjuvant

The results of solvent screening are shown in Table 1. The original pesticide could not be completely dissolved using a single solvent. However, 5% *N*-methyl-2-pyrrolidone + 10% cyclohexanone could completely dissolve the original pesticide. There was no solid precipitation at room temperature, so the formulation could be used for the subsequent experiment. According to Table 2, a mixture of sulfonate adjuvants (70b) and fatty alcohol polyoxyethylene ether adjuvants (AEO-4, -5, -7, -9, 992) could stabilize the system in a single, transparent, homogeneous phase. Therefore, sulfonate adjuvant (70b) was selected and mixed with five adjuvants of the AEO series to prepare thidiazuron·diuron ultra-low-volume sprays, numbered 1–5 (as shown in Table 3).

**Table 1:** Selection of solvent type and dosage (%: mass fraction).

Sample	CYC (%)	NMP (%)	DMF (%)	AN (%)	THN (%)	Phenomenon
1	10					−
2	10	5	1		1	++
3	10		1			−
4		5	1			−
5		5			1	−
6			1	1		−
7			1		1	−
8	10	5			1	+
9	10	5	1			++
10	10	5		1		+
11	10	5				+++
12	10			1		−
13	10		2		1	+
14		5		1		−

−, original pesticide hardly dissolved; +, original pesticide partially dissolved; ++, original pesticide mostly dissolved, or a solid precipitate formed after completely dissolving at room temperature; +++, original pesticide completely dissolved, and no solid precipitated at room temperature. NMP, *N*-Methyl-2-pyrrolidone; AN, acetonitrile; THN, tetrahydronaphthalene; CYC, cyclohexanone.

**Table 2:** Selection of adjuvants type and dosage (%: mass fraction).

Anionic adjuvant		Nonionic adjuvant		State
Type	Dosage (%)	Type	Dosage (%)	
Sulfate	8	Fatty alcohol polyoxyethylene ether	9	Stratification
	8	Fatty acid ester ether	7	Emulsification
	8	Isomeric alcohol ethers	8	Stratification
	8	Polyether-block polyether	8	Emulsification
Sulfonate	5	Fatty alcohol polyoxyethylene ether	7	Transparent homogeneous
	5	Fatty acid ester ether	9	Emulsification
	5	Isomeric alcohol ethers	8	Stratification
	5	Polyether-block polyether	8	Solid precipitation
Phosphate	9	Fatty alcohol polyoxyethylene ether	9	Stratification
	9	Fatty acid ester ether	7	Solid precipitation
	9	Isomeric alcohol ethers	8	Stratification
	9	Polyether-block polyether	8	Emulsification
Carboxylate	5	Fatty alcohol polyoxyethylene ether	9	Stratification
	5	Fatty acid ester ether	7	Emulsification
	5	Isomeric alcohol ethers	8	Solid precipitation
	5	Polyether-block polyether	8	Emulsification

**Table 3:** Ultra-low-volume formulations used in this study.

Sample	Organic solvent		Anionic adjuvant	Nonionic adjuvant	Oily solvent	Filler
1	NMP	CYC	70b	AEO-4	Methyl oleate	Chlorinated paraffin
2	NMP	CYC	70b	AEO-5	Methyl oleate	Chlorinated paraffin
3	NMP	CYC	70b	AEO-7	Methyl oleate	Chlorinated paraffin
4	NMP	CYC	70b	AEO-9	Methyl oleate	Chlorinated paraffin
5	NMP	CYC	70b	992	Methyl oleate	Chlorinated paraffin

### Surface tension measurement

**Figure 1:**
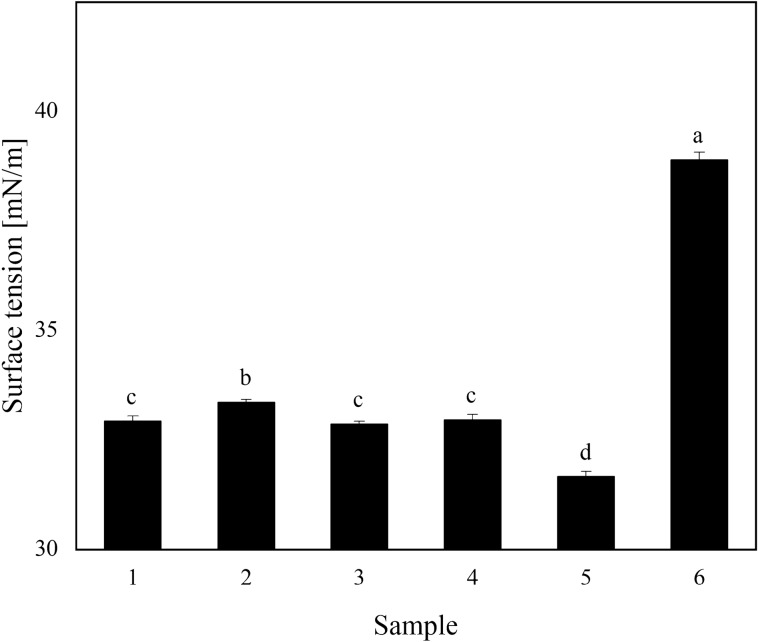
Surface tensions of different samples. Different letters (**a**–**d**) indicate significant differences between means. Means followed by the same letter are not significant at the 5% significance level by the LSD test (LSD = 0.05). Vertical bars indicate a standard deviation of the mean. The detailed data of the histogram is shown in Supplementary Table S1.

The critical surface tension of cotton leaves is 63.30–71.81 mN/m. Figure 1 shows that the surface tension of each sample was 31.67–33.37 mN/m, which was much lower than the critical surface tension of the leaf, indicating the agent was able to completely wet the leaf and be fully distributed on the leaf surface. The maximum surface tension of the reference product was 38.90 mN/m. Under the same dosage of adjuvant, sample 5 with adjuvant 992 had the smallest surface tension of 31.67 mN/m.

### Contact angle measurement

**Figure 2:**
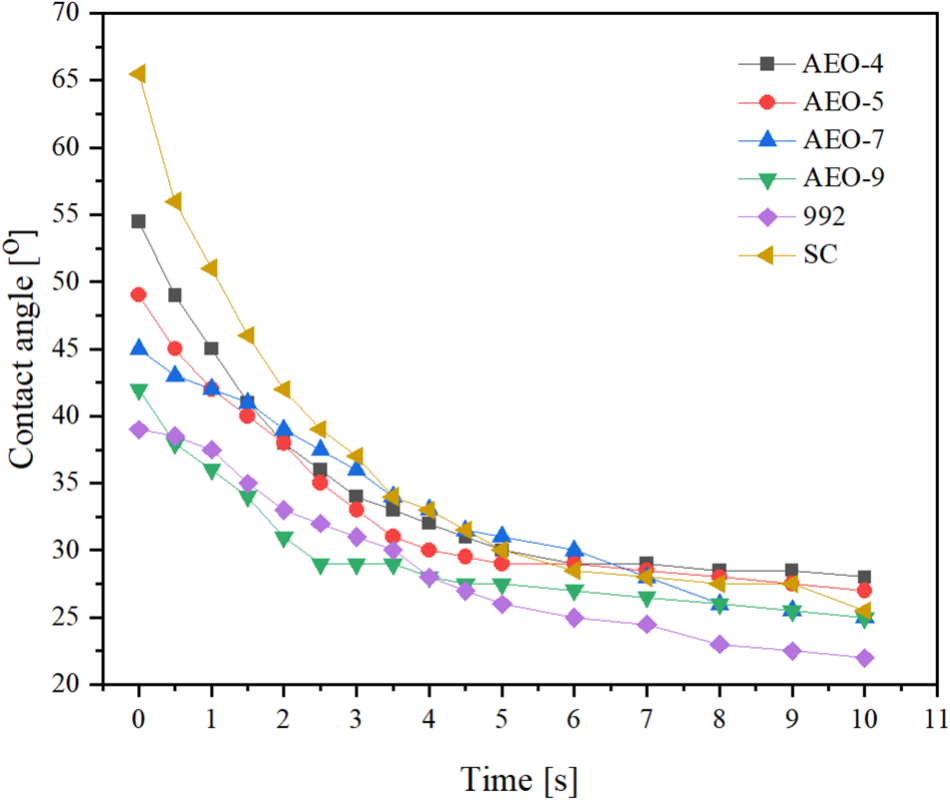
Contact angles of different samples on cotton leaves in 0–10 s. The detailed data of drawing the contact Angle curve is shown in Supplementary Table S2.

According to Young's equation, the smaller the surface tension, the smaller the contact angle^40,41^. Figure 2 shows the contact angle of different samples on cotton leaves and the change in contact angle over time. The contact angles of oil agents containing the adjuvant 992, AEO-7 and AEO-9 were smaller than that of the reference product, and the spreading effect was superior to that of the reference product. In the surface tension test, sample 5 had the smallest surface tension of 31.67 mN/m; this sample showed the minimum initial contact angle (39°) and a static contact angle (22°). The surface tension of the reference product was 38.90 mN/m., with the maximum initial contact angle (65.5°). Therefore, the relationship between surface tension and contact angle conformed to Young's equation.

### Volatilization rate measurement

**Figure 3:**
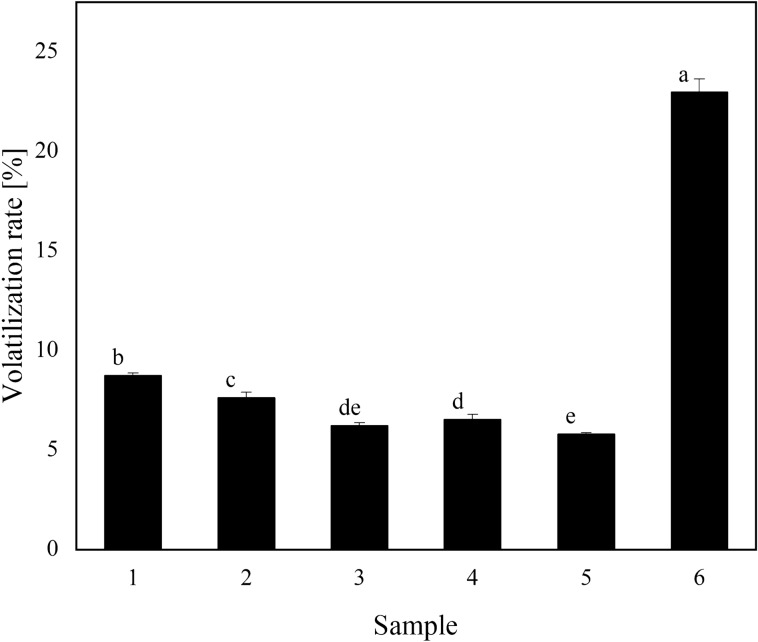
Volatilization of different samples on filter paper. Different letters (**a–e**) indicate significant differences between means. Means followed by the same letter are not significant at the 5% significance level by the LSD test (LSD = 0.05). Vertical bars indicate a standard deviation of the mean. The detailed data of the histogram is shown in Supplementary Table S3.

As shown in Fig. 3, the volatilization rate of the oil agent was much lower than that of the reference product. The volatilization rate of the five treatments was 5.80–8.74%, while the volatilization rate of the reference product was 22.97%. The volatilization rate of the oil agent met the quality requirements of an ultra-low-volume spray (≤ 30%). A low volatilization rate helps with spraying defoliants in hot and dry areas such as Xinjiang, effectively preventing evaporation of the droplets and increasing deposition.

### Viscosity measurement

**Figure 4:**
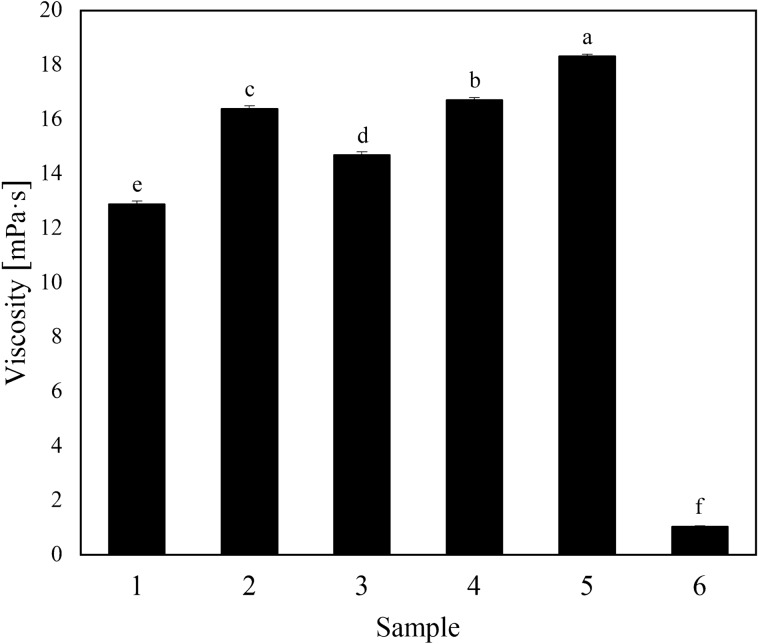
Viscosity of each sample. Different letters (**a–f**) indicate significant differences between means. Means followed by the same letter are not significant at the 5% significance level by the LSD test (LSD = 0.05). Vertical bars indicate a standard deviation of the mean. The detailed data of the histogram is shown in Supplementary Table S4.

Viscosity is an important factor affecting the atomization performance of a formulation^42^. Figure 4 shows that the viscosity of the five oil agents ranged from 12.9 to 18.3 mPa s, meeting the quality requirements of an ultra-low-volume spray (< 2 Pa s). The addition of chlorinated paraffin effectively increased the viscosity, facilitating analysis of the relationship between deposition and the viscosity of droplets in the subsequent experiment. This part of the work is described below under atomization performance.

### Screening for the best working conditions for the centrifugal spray atomizer

**Figure 5:**
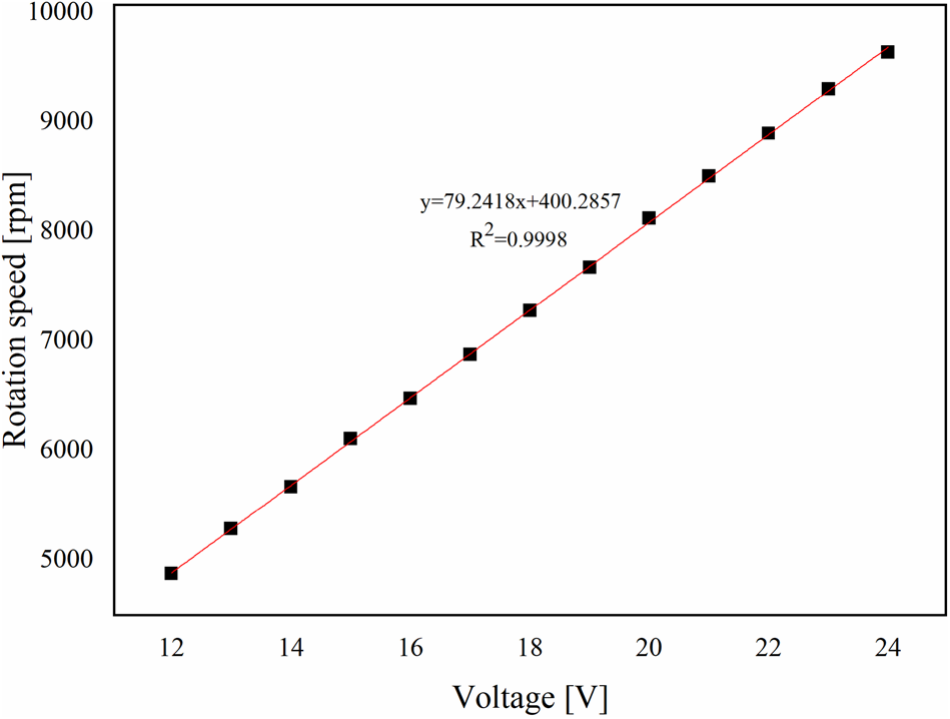
Relationship between voltage and rotation speed of the centrifugal spray atomizer. The detailed data of drawing the curve is shown in Supplementary Table S5.

As shown in Fig. 5, the linear equation fitted to voltage and rotation speed was y = 79.2418x + 400.2857, and the correlation coefficient was 0.9998. This indicated a good linear relationship and that the working state of the rotary atomizer was stable, and met the requirements of the next experiment.

**Figure 6:**
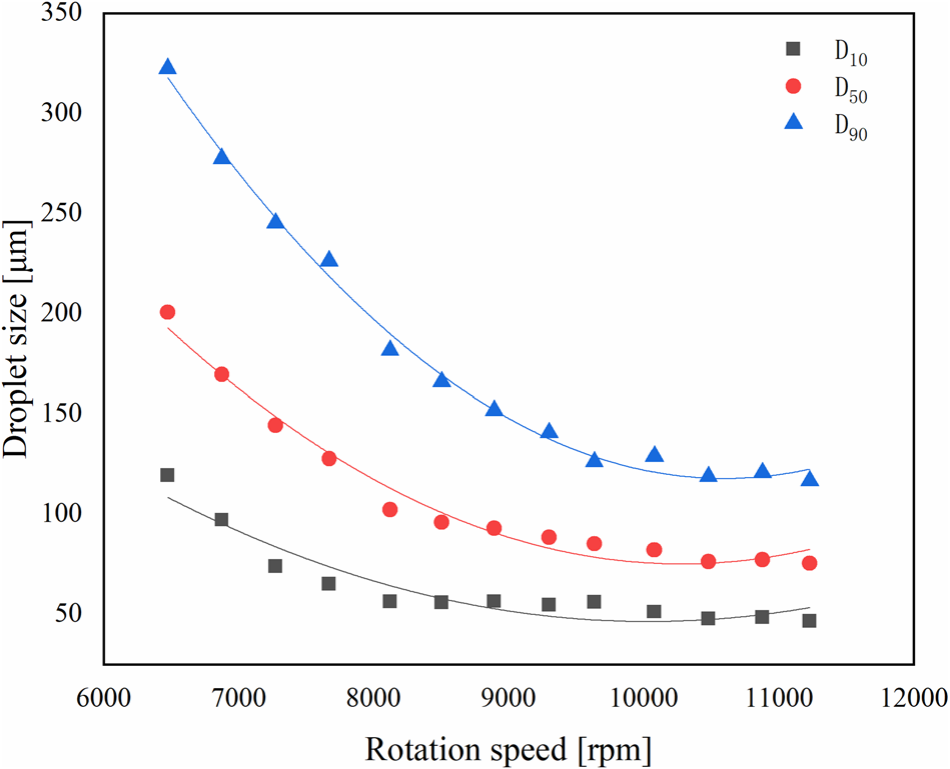
Relationship between the rotation speed of the centrifugal spray atomizer and droplet size. D_10_: 10% cumulative volume diameter, D_50_: 50% cumulative volume diameter, D_90_: 90% cumulative volume diameter. The detailed data of drawing the curve is shown in Supplementary Table S6.

**Figure 7:**
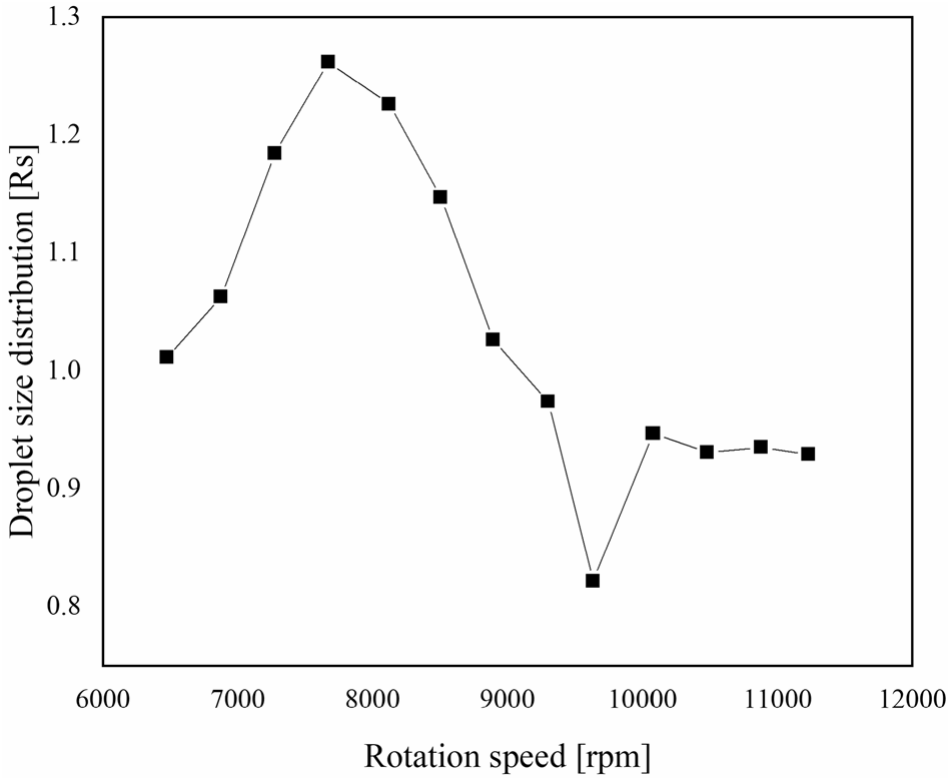
Relationship between the rotation speed of the centrifugal spray atomizer and the fog droplet spectrum. The detailed data of drawing the curve is shown in Supplementary Table S6.

The relationship between droplet size and the rotation speed of the rotary atomizer are shown in Fig. 6. The rotation speed of the rotary atomizer and the cumulative volume diameter had a binomial distribution. The equation fitted to D_10_ was y = 1E−05x^2^ − 0.2496x + 1440.4, with a correlation coefficient of 0.9947; the equation fitted to D_50_ was y = 8E−06x^2^ – 0.1672x + 934.73, with a correlation coefficient of 0.9791; the equation fitted to D_90_ was y = 5E−06x^2^ – 0.0983x + 539.55, with a correlation coefficient of 0.9005. The correlation between the cumulative volume diameter and the rotation speed was very high, and the cumulative volume diameter became smaller as the rotation speed increased. When the rotation speed exceeded 9600 rpm (voltage 20 V), the cumulative volume diameter hardly changed. As shown in Fig. 7, when the rotation speed was 6400–7600 rpm (voltage 10–15 V), the spectral width of droplets increased with increasing rotation speed, and the distribution of droplets became more uneven. When the rotation speed was 7600–9600 rpm (voltage 15–20 V), the spectral width of droplets decreased with increasing rotation speed, and the distribution of droplets became more uniform. The minimum droplet size distribution appeared at 9600 rpm (voltage 15–20 V). When the rotation speed surpassed 9600 rpm (voltage > 20 V), the droplet size distribution tended to be stable. This coincided with data shown in Fig. 6, where the inflection point appeared when rotation speed was 9600 rpm (voltage = 20 V).

Therefore, we determined that the optimal working conditions for the rotary atomizer were achieved by setting the DC voltage stabilized power supply current to 1.00 A and voltage to 20 V, which were used for subsequent experiments.

### Atomization performance

**Figure 8:**
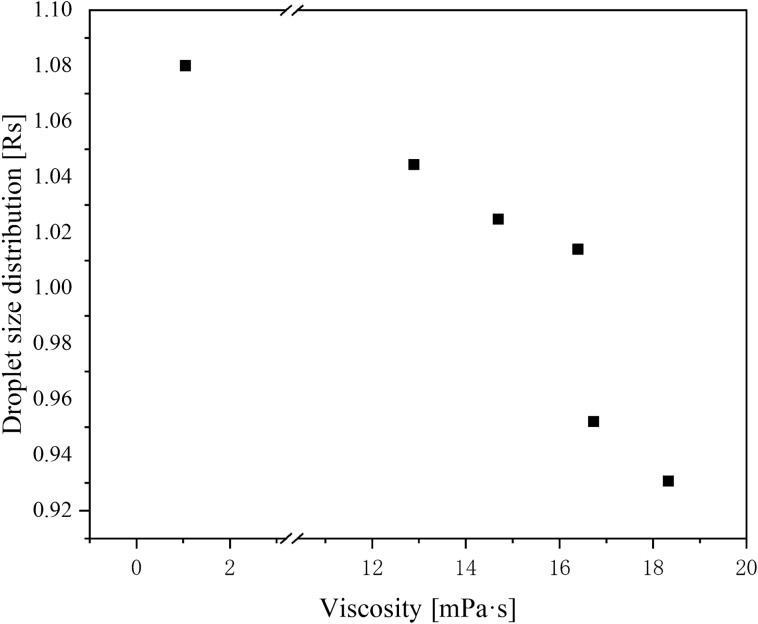
Relationship between formulation viscosity and droplet spectrum. The detailed data of drawing the figure is shown in Supplementary Table S7.

The relationship between viscosity and droplet spectrum are shown in Table 4 and Fig. 8. The cumulative volume diameter for the five treatments was less than 150 μm meeting the requirements of the ULV spray^32^. The cumulative volume diameter for the five treatments was larger than that for the reference product, the width of the droplet spectrum was narrower, and the droplet distribution was more uniform. Droplet size affects the drift of droplets^43^. The D_10_ of the reference product was 25.62 μm under these working conditions. This droplet size was highly susceptible to drift and deposition on non-target organisms. Water suspension was not suitable for this application at low dosage.

**Table 4:** Droplet size and droplet size distribution of different sample sprays.

Sample	Droplet size (μm)			Rs	Viscosity (mPa s)
	D_10_	D_50_	D_90_		
1	48.99	82.74	134.11	1.03	12.90 ± 0.10e
2	53.32	86.34	142.31	1.03	16.40 ± 0.10c
3	51.09	83.92	138.33	1.04	14.70 ± 0.10d
4	53.63	86.93	144.90	1.05	16.73 ± 0.06b
5	55.25	91.98	148.87	1.02	18.33 ± 0.06a
6	25.62	56.19	107.85	1.46	1.05 ± 0.15f

D_10_: 10% cumulative volume diameter, D_50_: 50% cumulative volume diameter, D_90_: 90% cumulative volume diameter, Rs: the droplet size distribution. Different letters (a, b, c, d, e, and f) indicate significant differences between means. Means followed by the same letter are not significant at the 5% significance level by the LSD test (LSD = 0.05). A number that follows the ± sign is a standard deviation (s.d.)

As presented in Table 4, droplet size increased with increasing viscosity, which influenced the droplet spectrum. The results in Fig. 8 show that the span of droplet size decreased with the increase of viscosity, indicating that droplets with more uniform distribution could be obtained by increasing the viscosity of the formulation^41^.

### Droplet deposition effect

We tested the efficacy of the ULV spray formulation by spraying cotton plants using an UAV. The test results in Table 5 indicate that increasing the dosage of application would increase droplet size, coverage, and deposition density. At the same application dosage, the droplet size of the ultra-low-volume spray was slightly larger than that of the reference product, and the coverage and deposition density were greater than those of the reference product. The droplet spectral width (Rs) of the five treatments was less than 1, and the coefficient of variation was less than 7%, indicating that the droplet distribution was relatively uniform. Among treatments, T2 had the narrowest Rs and coefficient of variation (CV), where the droplet size distribution was the most uniform. For the ultra-low-volume spray, at the application dosage of 4.5–9.0 L/ha, the droplet coverage gradually increased from 0.85 to 4.15%; the droplet deposition densities were 15.63, 17.24, 28.45, and 42.57 pcs/cm^2^, which were larger than requirements suggested in the literature. The droplet coverage of the reference product (T5) was 0.73%, and the deposition density was only 11.32 pcs/cm^2^.

**Table 5:** Droplet size, coverage, deposition density, spectral width and variation coefficient for each treatment.

Treatment	Application amount (L/ha)	Droplet size (μm)			Coverage (%)	Deposition density (pcs/cm^2^)	Rs	Variation coefficient (%)
		D_10_	D_50_	D_90_				
T1	4.50	92	134	202	0.85 ± 0.09c	15.63 ± 1.60c	0.82	5.73
T2	6.00	98	159	211	0.91 ± 0.11c	17.24 ± 1.08c	0.71	3.46
T3	7.50	105	172	235	1.87 ± 0.34b	28.45 ± 2.55b	0.75	3.58
T4	9.00	114	201	276	4.15 ± 0.39a	42.57 ± 2.31a	0.81	6.72
T5	6.00	86	124	193	0.73 ± 0.11c	11.32 ± 1.94d	0.86	5.74

Different letters (a, b, c, and d) indicate significant differences between means. Means followed by the same letter are not significant at the 5% significance level by the LSD test (LSD = 0.05). A number that follows the ± sign is a standard deviation (s.d.)

### Efficacy trials

**Figure 9:**
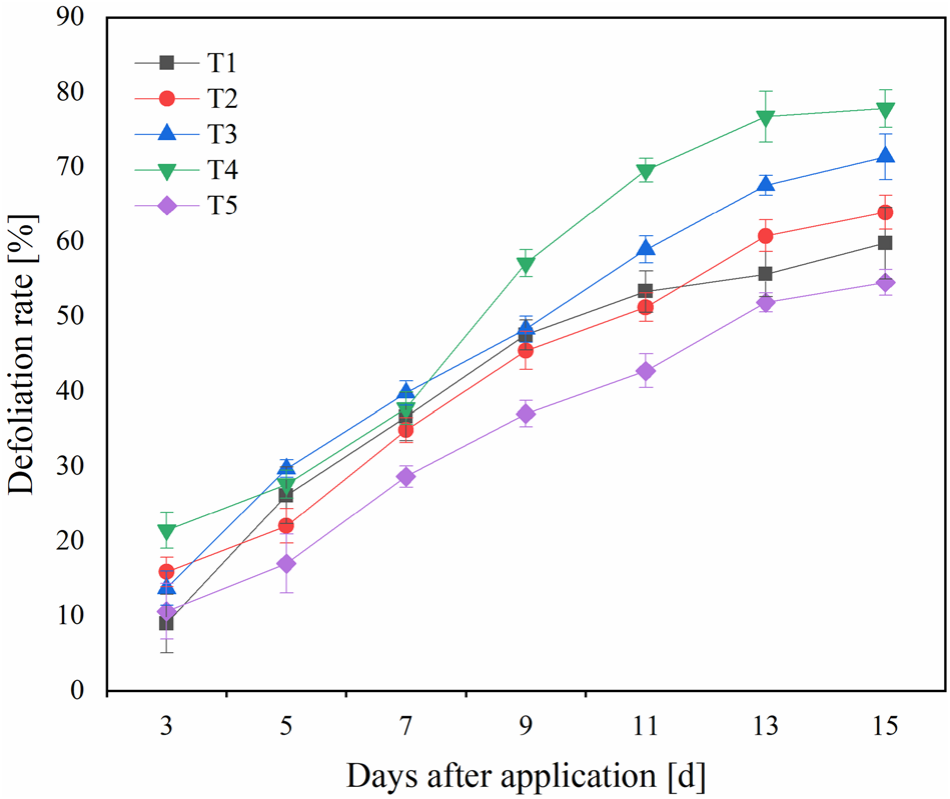
Defoliation rate 3–15 days after treatment. The detailed data of drawing the curve is shown in Supplementary Table S8.

**Figure 10:**
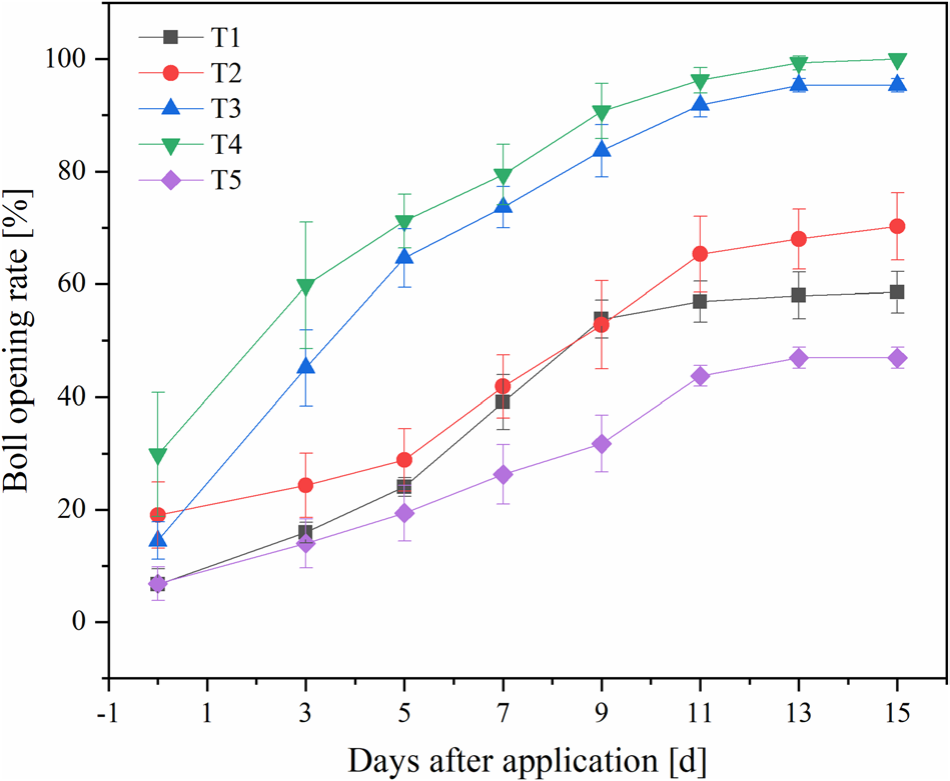
Boll opening rate 3–15 days after treatment. The detailed data of drawing the curve is shown in Supplementary Table S9.

The efficacy of cotton defoliant is reflected in the defoliation rate and boll opening rate of cotton after application. Therefore, we surveyed the defoliation rate and boll opening rate of cotton in the test area 3–15 days after application. The results are shown in Figs. 9 and 10.

Figure 9 indicates that the defoliation rates of the five treatments 15 days after the pesticide treatment were 59.82%, 63.96%, 71.40%, 77.84%, and 54.58%, respectively. The defoliation rates of T1, T2, and T5 were less than 70%.

Application of the ultra-low-volume spray at 4.50 L/ha or 6.00 L/ha and the reference product at 6.00 L/ha had a poor defoliation effect. T4 (9.00 L/ha) was superior to the others, and the defoliation rate reached 77.84% 15 days after application. As shown in Fig. 10, the boll opening rates of the five treatments were 58.54%, 67.74%, 95.35%, 100%, and 44.68% 15 days after application. Similarly, the boll opening rates of T1, T2, and T5 were poor, with the boll opening rate of the control T5 only 44.68%. We analyzed significant differences between the defoliation rates and boll opening rates of the five treatments. The results showed that the defoliation rate and boll opening rate associated with the thidiazuron·diuron ultra-low-volume spray on cotton plants were significantly different from those of the reference product.

Overall, the defoliation rate and boll opening rate produced by the ultra-low-volume spray were superior to those produced by the reference product. This result was consistent with data shown in Table 5. The higher the droplet coverage rate, the higher the droplet deposition density and the higher the defoliation rate and boll opening rate. T1, T2 and T5 had poor deposition effect on cotton plants, and the effective pesticide utilization rate was low, resulting in dissatisfactory defoliation rates and boll opening rates. Both the droplet coverage rate and the droplet deposition density of T3 and T4 were large. Therefore, droplets of pesticide solution could deposit more easily and uniformly on cotton leaves, allowing the plants to defoliate and open their bolls easily.

## Conclusions

We prepared a thidiazuron·diuron ultra-low-volume spray that met the quality requirements of an ultra-low-volume spray. We evaluated the application of thidiazuron·diuron ultra-low-volume spray compared with the reference product, 54% tidiazuron·diuron suspension concentrate. The surface tension of the oil agent was lower, the initial contact angle and the static contact angle on cotton leaves were smaller, and the spreading speed was faster than those of the reference product, indicating that the oil agent had good wetting and spreading performance. At the same time, the volatilization rate of ultra-low-volume sprays was much lower than that of the reference product, which was beneficial to use in hot and dry areas such as Xinjiang.

We determined the optimal working conditions of the rotary atomizer and tested the atomization performance of the formulations using an indoor spraying device. The cumulative volume particle size of the ultra-low-volume spray was larger than that of the reference product, and the droplet size distribution was more uniform. In addition, droplet size increased with increase in viscosity while droplet size spectrum decreased with increase in viscosity, indicating that droplets with more uniform distribution can be obtained by increasing the viscosity of the formulation.

We examined the deposition effect of the formulation, as well as the defoliation rate and boll opening rate of cotton, in an efficacy trial. Droplet size, coverage rate, and deposition density increased with increased dosage. Under the same dosage, the droplet size of the ultra-low-volume spray was slightly larger than that of the reference product, and the coverage rate and deposition density were greater than those of the reference product. The ultra-low-volume spray at 9.00 L/ha showed the best defoliation effect at 15 days after application, with a defoliation rate of 77.84% and boll opening rate of 100%. The defoliation rate of the reference product was 54.58%, and the boll opening rate was only 44.68%. Therefore, ultra-low-volume spray could be considered as an ideal pesticide formulation suitable for plant protection UAV. It had the characteristics of low dosage, high efficiency and environmentally friendly.

## Supplementary Information

Supplementary Information.

## Data Availability

Data is contained within the article or Supplementary Information.
