# Supplementary material for: Preparation and application of a thidiazuron·diuron ultra-low-volume spray suitable for plant protection unmanned aerial vehicles
Source: Sci Rep. 2021 Mar 2;11:4998. doi: 10.1038/s41598-021-84459-4 (PMC7925647; doi:10.1038/s41598-021-84459-4)
Supplement: Supplementary file 1 — Supplementary Information. [file 41598_2021_84459_MOESM1_ESM.docx]

Supplementary Material for

**Preparation and application of a thidiazuron·diuron ultra-low-volume spray suitable for plant protection unmanned aerial vehicles**

Qin Liu1,+, Kun Wei2,+, Liyun Yang^1^, Weiming Xu1,* & Wei Xue1,*

1State Key Laboratory Breeding Base of Green Pesticide and Agricultural Bioengineering, Key Laboratory of Green Pesticide and Agricultural Bioengineering, Ministry of Education, Center for Research and Development of Fine Chemicals, Guizhou University, Guiyang, 550025, China.

2Renhuai Agricultural and Rural Bureau, Guizhou, Renhuai, 564500, China.

*Corresponding author: wmxu@gzu.edu.cn (Weiming Xu); wxue@gzu.edu.cn (Wei Xue)

+These authors contributed equally to this work.

**Table Legends**

**Table S1.** Surface tension of different samples.

**Table S2.** Contact angle of different samples (°).

**Table S3.** Volatilization of different samples on filter paper.

**Table S4.** Viscosity of different samples.

**Table S5.** Relationship between voltage and rotation speed of the centrifugal spray atomizer.

**Table S6.** Relationship between the rotation speed of centrifugal spray atomizer and droplet size. Relationship between the rotation speed of the centrifugal spray atomizer and the fog droplet spectrum.

**Table S7.** Relationship between formulation viscosity and droplet spectrum.

**Table S8.** Defoliation rate 3-15 days after treatment.

**Table S9.** Boll opening rate 3-15 days after treatment.

**Table S1.** Surface tension of different samples.

|  | Surface tension (mN/m) | | | Average |
| --- | --- | --- | --- | --- |
|  | 1st | 2nd | 3rd |  |
| 1 | 32.80 | 33.00 | 33.00 | 32.93±0.12c |
| 2 | 33.30 | 33.40 | 33.40 | 33.37±0.06b |
| 3 | 32.90 | 32.80 | 32.90 | 32.87±0.06c |
| 4 | 33.10 | 32.90 | 32.90 | 32.97±0.12c |
| 5 | 31.60 | 31.60 | 31.80 | 31.67±0.12d |
| 6 | 38.80 | 39.10 | 38.80 | 38.90±0.18a |

Different letters (a, b, c, and d) indicate significant differences between means. Means followed by the same letter are not significant at the 5% significance level by the LSD test (LSD = 0.05). A number that follows the ± sign is a standard deviation (s.d.).

**Table S2.** Contact angle of different samples (°).

| Sample | 0 s | 0.5s | 1 s | 1.5 s | 2 s | 2.5 s | 3 s | 3.5 s | 4 s | 4.5 s | 5 s | 6 s | 7 s | 8 s | 9 s | 10 s |
| --- | --- | --- | --- | --- | --- | --- | --- | --- | --- | --- | --- | --- | --- | --- | --- | --- |
| 1 | 54.5 | 49 | 45 | 41 | 38 | 36 | 34 | 33 | 32 | 31 | 30 | 29 | 29 | 28.5 | 28.5 | 28 |
| 2 | 49 | 45 | 42 | 40 | 38 | 35 | 33 | 31 | 30 | 29.5 | 29 | 29 | 28.5 | 28 | 27.5 | 27 |
| 3 | 45 | 43 | 42 | 41 | 39 | 37.5 | 36 | 34 | 33 | 31.5 | 31 | 30 | 28 | 26 | 25.5 | 25 |
| 4 | 42 | 38 | 36 | 34 | 31 | 29 | 29 | 29 | 28 | 27.5 | 27.5 | 27 | 26.5 | 26 | 25.5 | 25 |
| 5 | 39 | 38.5 | 37.5 | 35 | 33 | 32 | 31 | 30 | 28 | 27 | 26 | 25 | 24.5 | 23 | 22.5 | 22 |
| 6 | 65.5 | 56 | 52 | 46 | 42 | 39 | 37 | 34 | 33 | 31.5 | 30 | 28.5 | 28 | 27.5 | 27.5 | 25.5 |

**Table S3.** Volatilization of different samples on filter paper.

| Sample | m_1_ (mg) | m_2_ (mg) | m_3_ (mg) | Volatilization (%) | Average (%) |
| --- | --- | --- | --- | --- | --- |
| 1 | 1.1252 | 2.0820 | 1.9975 | 8.74 | 8.74±0.12b |
|  | 1.1248 | 2.0791 | 1.997 | 9.60 |  |
|  | 1.1339 | 2.1078 | 2.0224 | 8.79 |  |
| 2 | 1.1339 | 1.9862 | 1.9217 | 7.57 | 7.63±0.27c |
|  | 1.1315 | 1.9839 | 1.9209 | 7.39 |  |
|  | 1.1289 | 2.0727 | 1.9979 | 7.93 |  |
| 3 | 1.1413 | 2.1323 | 2.0702 | 6.27 | 6.23±0.13de |
|  | 1.1329 | 2.1249 | 2.0645 | 6.09 |  |
|  | 1.1268 | 1.9323 | 1.8813 | 6.33 |  |
| 4 | 1.1386 | 1.9376 | 1.8858 | 6.48 | 6.53±0.25d |
|  | 1.1289 | 1.8905 | 1.8387 | 6.80 |  |
|  | 1.1224 | 1.8868 | 1.8385 | 6.32 |  |
| 5 | 1.1352 | 1.9076 | 1.8634 | 5.72 | 5.80±0.07e |
|  | 1.1298 | 1.8445 | 1.8027 | 5.85 |  |
|  | 1.1268 | 1.8423 | 1.8006 | 5.83 |  |
| 6 | 1.1286 | 2.1123 | 1.8843 | 23.18 | 22.97±0.66a |
|  | 1.1298 | 2.1119 | 1.8935 | 22.24 |  |
|  | 1.1365 | 2.1232 | 1.8913 | 23.50 |  |

Different letters (a, b, c, d, and e) indicate significant differences between means. Means followed by the same letter are not significant at the 5% significance level by the LSD test (LSD = 0.05). A number that follows the ± sign is a standard deviation (s.d.).

**Table S4.** Viscosity of different samples.

| Sample | Viscosity (mPa. s) | | | Average |
| --- | --- | --- | --- | --- |
|  | 1st | 2nd | 3rd |  |
| 1 | 12.90 | 12.80 | 13.00 | 12.90±0.10e |
| 2 | 16.30 | 16.40 | 16.50 | 16.40±0.10c |
| 3 | 14.60 | 14.80 | 14.70 | 14.70±0.10d |
| 4 | 16.70 | 16.80 | 16.70 | 16.73±0.06b |
| 5 | 18.30 | 18.40 | 18.30 | 18.33±0.06a |
| 6 | 1.04 | 1.05 | 1.07 | 1.05±0.02f |

Different letters (a, b, c, d, e and f) indicate significant differences between means. Means followed by the same letter are not significant at the 5% significance level by the LSD test (LSD = 0.05). A number that follows the ± sign is a standard deviation (s.d.).

**Table S5.** Relationship between voltage and the rotation speed of centrifugal spray atomizer.

| Voltage (V) | 12 | 13 | 14 | 15 | 16 | 17 | 18 | 19 | 20 | 21 | 22 | 23 | 24 |
| --- | --- | --- | --- | --- | --- | --- | --- | --- | --- | --- | --- | --- | --- |
| Rotation speed (rpm) | 4874 | 5286 | 5668 | 6108 | 6475 | 6876 | 7275 | 7672 | 8123 | 8506 | 8895 | 9302 | 9637 |

**Table S6.** Relationship between the rotation speed of the centrifugal spray atomizer and droplet size. Relationship between the rotation speed of the centrifugal spray atomizer and the fog droplet spectrum.

| Rotation speed (rpm) | Droplet size (μm) | | | Rs |
| --- | --- | --- | --- | --- |
|  | D_10_ | D_50_ | D_90_ |  |
| 6475 | 119.35 | 200.64 | 322.31 | 1.01156 |
| 6876 | 97.08 | 169.65 | 277.37 | 1.06272 |
| 7275 | 74.04 | 144.42 | 245.09 | 1.18439 |
| 7672 | 65.11 | 127.63 | 226.16 | 1.26185 |
| 8123 | 56.26 | 102.25 | 181.62 | 1.22601 |
| 8506 | 55.8 | 95.97 | 165.89 | 1.14713 |
| 8895 | 56.42 | 92.92 | 151.79 | 1.02637 |
| 9302 | 54.58 | 88.4 | 140.69 | 0.9741 |
| 9637 | 56.15 | 85.23 | 126.19 | 0.82178 |
| 10079 | 51.18 | 82.1 | 128.91 | 0.94677 |
| 10479 | 47.77 | 76.31 | 118.79 | 0.93068 |
| 10879 | 48.44 | 77.38 | 120.8 | 0.93513 |
| 11229 | 46.62 | 75.46 | 116.74 | 0.92923 |

D_10_: 10% cumulative volume diameter, D_50_: 50% cumulative volume diameter, D_90_: 90% cumulative volume diameter, Rs: the droplet size distribution.

**Table S7.** Relationship between formulation viscosity and droplet spectrum.

| Sample | Droplet size (μm) | | | Rs | Viscosity (mPa. s) |
| --- | --- | --- | --- | --- | --- |
|  | D_10_ | D_50_ | D_90_ |  |  |
| 1 | 70.26 | 92.74 | 167.11 | 1.04 | 12.9 |
| 2 | 74.63 | 96.34 | 172.31 | 1.01 | 16.4 |
| 3 | 73.09 | 93.92 | 169.33 | 1.02 | 14.7 |
| 4 | 76.63 | 96.93 | 168.90 | 0.95 | 16.73 |
| 5 | 80.25 | 105.98 | 178.87 | 0.93 | 18.33 |
| 6 | 56.75 | 84.56 | 148.07 | 1.08 | 1.05 |

D_10_: 10% cumulative volume diameter, D_50_: 50% cumulative volume diameter, D_90_: 90% cumulative volume diameter, Rs: the droplet size distribution.

**Table S8.** Defoliation rate 3-15 days after treatment.

| Days after application | Defoliation rate (%) | | | | |
| --- | --- | --- | --- | --- | --- |
|  | T1 | T2 | T3 | T4 | T5 |
| 3 | 6.07143 | 18.27411 | 15.20147 | 24.25629 | 6.22711 |
| 5 | 23.75 | 24.8731 | 31.13553 | 28.83295 | 12.82051 |
| 7 | 32.32143 | 36.20981 | 40.29304 | 36.84211 | 28.57143 |
| 9 | 46.78571 | 48.22335 | 48.1685 | 56.29291 | 36.44689 |
| 11 | 50.35714 | 52.79188 | 59.26773 | 69.96337 | 40.47619 |
| 13 | 57.85714 | 63.95939 | 69.33638 | 76.73993 | 53.2967 |
| 15 | 59.82143 | 63.95939 | 71.39588 | 77.83883 | 54.57875 |

**Table S9.** Boll opening rate 3-15 days after treatment.

| Days after application | Boll opening rate (%) | | | | |
| --- | --- | --- | --- | --- | --- |
|  | T1 | T2 | T3 | T4 | T5 |
| 0 | 4.06504 | 20.96774 | 10.46512 | 47.82609 | 3.19149 |
| 3 | 16.26016 | 25.80645 | 54.65116 | 71.73913 | 8.51064 |
| 5 | 23.57724 | 29.83871 | 69.76744 | 76.91304 | 14.89362 |
| 7 | 39.8374 | 43.54839 | 77.90698 | 84.08696 | 20.21277 |
| 9 | 53.65854 | 53.22581 | 89.53488 | 97.82609 | 25.53191 |
| 11 | 57.72358 | 66.93548 | 94.18605 | 100 | 43.61702 |
| 13 | 58.53659 | 67.74194 | 95.34884 | 100 | 44.68085 |
| 15 | 58.53659 | 67.74194 | 95.34884 | 100 | 44.68085 |
